# Using the *Pf*EMP1 Head Structure Binding Motif to Deal a Blow at Severe Malaria

**DOI:** 10.1371/journal.pone.0088420

**Published:** 2014-02-07

**Authors:** Manuel E. Patarroyo, Martha Patricia Alba, Hernando Curtidor, Magnolia Vanegas, Hannia Almonacid, Manuel A. Patarroyo

**Affiliations:** 1 Fundación Instituto de Inmunología de Colombia (FIDIC), Bogotá, Colombia; 2 School of Medicine, Universidad Nacional de Colombia, Bogotá, Colombia; 3 School of Medicine and Health Sciences, Universidad del Rosario, Bogotá. Colombia; National Institutes of Health, United States of America

## Abstract

*Plasmodium falciparum* (*Pf*) malaria causes 200 million cases worldwide, 8 million being severe and complicated leading to ∼1 million deaths and ∼100,000 abortions annually. *Plasmodium falciparum* erythrocyte membrane protein 1 (*Pf*EMP1) has been implicated in cytoadherence and infected erythrocyte rosette formation, associated with cerebral malaria; chondroitin sulphate-A attachment and infected erythrocyte sequestration related to pregnancy-associated malaria and other severe forms of disease. An endothelial cell high activity binding peptide is described in several of this ∼300 kDa hypervariable protein’s domains displaying a conserved motif (GACxPxRRxxLC); it established H-bonds with other binding peptides to mediate red blood cell group A and chondroitin sulphate attachment. This motif (when properly modified) induced *Pf*EMP1-specific strain-transcending, fully-protective immunity for the first time in experimental challenge in *Aotus* monkeys, opening the way forward for a long sought-after vaccine against severe malaria.

## Introduction

Malaria-infected children’s sera originally recognised *Pf*EMP1 in infected erythrocyte (IE) agglutination tests [1], as a highly polymorphic very large (∼300 kDa molecular weight); protein encoded by >60 variable, genes (*Pf var*). *Pf*EMP1 has an extracellular ectodomain consisting of 2 to 9 highly variable in amino acid sequence, length and composition domains; constituted by an N-terminal segment (NTS), a Duffy-binding-like (DBL) 1α domain and a cysteine interdomain region (CIDR) α1 (forming the head structure) and DBL2X, C2, DBL3X, DBL4ε, DBL5ε, DBL6ε and DBL7ε domains followed by a transmembrane region (TM), and an intracytoplasmic acidic terminal segment (ATS), inserted into IE membrane [2]–[4].

*Pf*EMP1 can be classified into 5 groups (A–E) based on the nucleotide sequence similarity of the upstream promoter sequence (UPS) [5], having 6 major DBL domain classes (α, β, γ, δ, ε and X). Each DBL domain consist of hypervariable and conserved regions and contains 3 subdomains (S1, S2 and S3) having 10 semi-conserved homology blocks (HB 1–10 consisting of 7 to 21 residues) conserved in all domain classes, most frequently localised in subdomains S1 (HB4), S2 (HB3, HB5) and S3 (HB2, HB1) [5], [6].

*Pf*EMP1 can also be grouped according to 23 domain cassettes (DC), the most frequent ones DC1 to 3, spanning the entire protein while the others include 2–4 domains [6].

The DBLα1 domain, binds blood group A and forms rosettes by adhering to uninfected erythrocytes (UE) [7] being associated with cerebral malaria (CM) [8]. DBL3X and DBL6ε bind to chondroitin sulphate proteoglycans (CSPG) whilst DBL2X, DBL3X, DBL5ε and DBL6ε bind to chondroitin sulphate-A (CSA) [9], [10], leading to IE sequestration in the placenta, thereby inducing pregnancy-associated malaria (PAM) and abortions, mainly in primigravidas.

A robust, highly specific, sensitive functional methodology has been thoroughly described for tailor-made vaccine development aimed at *Pf*EMP1 (*ipso facto* severe malaria), recognising variable and conserved HABPs (cHABPs) in relevant invasion molecules by working with ∼15 to 20 mer-long peptides [11]. cHABPs are immunologically silent since they do not induce immune responses; however, when their critical binding residues have been properly modified [12]–[14] they become highly immunogenic and protection-inducing modified HABPs (mHABPs).

## Materials and Methods

### Ethics Statement

The present study was approved by the Fundación Instituto de Inmunología’s animal ethics committee. The capture of *Aotus* monkeys (International Union for Conservation of Nature and Natural Resources (IUCN) status: least concern), the pertinent maintenance, immunization challenge and research procedures have been authorized by the official Colombian environmental authority in the Amazonian region (CORPOAMAZONIA, resolutions 0066/Sep/2006, 0028/May/2010, 0632/Jun/2010 and 0042/Jan/2011 and previous authorizations beginning in 1982).

The US Committee on the Care and Use of Laboratory Animals’ guidelines were followed for all animal handling procedures, in turn complying with Colombian regulations for biomedical research (resolution 8430/1993 and law 84/1989). Monkeys at the station were numbered, sexed, weighed, given a physical-clinical exam and kept temporally in individual cages, prior to all experimental procedures. They were kept in controlled conditions regarding temperature (25°–30° centigrade) and relative humidity (83%), similar to those present in their natural environment. The monkeys’ diet was based on a supply of fruit typical of the amazon region (i.e. such primates’ natural diet), vegetables and a nutritional supplement including vitamins, minerals and proteins. Environmental enrichment included visual barriers to avoid social conflict, feeding devices, some branches and vegetation, perches and habitat. Any procedure requiring animal handling was practiced by trained veterinary personnel and animals were submitted to sedation and analgesia procedures to reduce stress when necessary [15]. The monkeys were cared for by expert veterinarians and biologists and supervised weekly by CORPOAMAZONIA veterinarians.

All individuals were released back into the Amazon jungle after the experimental procedures and 30–40 days of quarantine and clinical evaluation in optimal health conditions, as approved by CORPOAMAZONIA and in the presence of its officials.

### Peptide Synthesis and Radiolabelling

All peptides were synthesised using standard *t*-Boc solid-phase peptide synthesis (SPPS) strategy [16]. A tyrosine residue was added to the C-terminus of peptides lacking it to allow radiolabelling, as widely described [14].

Polymeric peptides were obtained for immunisation purposes by adding CG to N- and -C termini, as previously described [14].

### Binding Assays with *Pf*EMP1 Peptides

*Pf*EMP1 binding to endothelial cells (C32 cells) and RBC was performed according to previously described protocols [14]. Peptides having binding activity greater than or equal to 2% (0.02 ratio) were considered high-activity binding peptides (HABPs), according to previously-established criteria [11].

### Animals and Immunisation

Groups of 4–10 Aotus monkeys proving IFA negative for *P. falciparum* blood stage, kept in our monkey colony in the Amazon jungle (Leticia, Colombia) according to National Institute of Health guidelines for animal handling and Colombia Ministry of Health laws (resolution 8430 of 1993 and law 84 of 1989) and directly supervised by CORPOAMAZONIA officials [17] and legal permits and authorization for capture and housing by the Colombian Ministry of the Environment have been in force for more than 30 years and there has been strong collaboration with the Colombian Association of Indian Authorities (ATICOYA, ASITAM and AZCAITA, representing ∼40 Indian communities) (pertinent documentation available on request), CORPOAMAZONIA 0266 (Dec/2010) being the most recent authorization.

*Aotus* monkeys were subcutaneously immunised twice or three times with 250 µg polymerised peptide (on days 1, 20 and 40) which had been previously homogenised with Freund’s complete adjuvant for the 1^st^ dose and Freund’s incomplete adjuvant for the 2^nd^ and 3^rd^ doses. Controls received only Freund’s adjuvant and saline solution on the same days. Blood samples were taken on day 1 before (P_0_) the first immunisation and 20 days after the 2^nd^ (II_20_) and 3^rd^ (III_20_) immunisations for immunological analysis [17].

### *Pf*EMP1 Detection by Immunofluorescence

Modifications of Staalsoe’s method (Cytrometry 35∶329) were used. Late trophozoite- and schizont-enriched FCB-2 *P. falciparum* cultures (5–10% parasitaemia) or highly infected *Aotus-*adapted *P. falciparum* FVO strain-enriched schizonts or late trophozoites were spun for 5 min at 1,800 rpm and left for a further 20 min to form sediment, washed three to four times with Tris-buffered Hanks’ solution (TBH) (10 ml 0.15M Tris buffer, pH 7.2, 90 ml 0.9% NaCl, and 100 ml Hanks’ solution) and then diluted to give 1% suspension.

Samples were sequentially treated for 15 min with 200 µl of the appropriate immune serum dilution followed by an anti-goat anti-*Aotus* IgG F (ab) 2 fragment conjugated with fluorescein isothiocyanate. Slides were washed with TBH supplemented with 50 ll Tween 20 per 100 ml between each sequential incubation. All incubations were performed at room temperature in a humidified chamber. Monolayers were counterstained by adding one drop of ethidium bromide per well to enable parasitised erythrocytes to be visualised. After a few seconds, slides were washed with distilled water, mounted and read at 100x in oil immersion.

### Western Blot Analysis

FVO strain culture *Pf*-schizont lysate was electrophoretically separated and transferred to nitrocellulose membranes. Each nitrocellulose strip was individually incubated with *Aotus* monkey sera diluted 1∶200 in blocking solution, washed several times and incubated with goat anti-*Aotus* IgG, F(ab) 2 fragment alkaline phosphatase (AP) conjugated at 1∶1,000 dilution and developed with NBT/BCIP [18].

### Challenge and Parasitaemia Assessment

Immunised and control *Aotus* monkeys were intravenously infected 20 days after the last immunisation with 100,000 *P. falciparum* FVO-strain infected RBC, a dose known to be 100% infective for these monkeys [17].

Protection was defined as the complete absence of parasites in blood during the 15 days of the experiment. Non-protected monkeys developed patent parasitaemia on day 5 or 6, reaching >5% levels between days 8 and 10. They then received treatment with antimalarial drugs and were kept in quarantine until ensuring complete cure, to be returned into the jungle later on [17].

Parasitaemia was measured daily for each monkey, starting on day 5 after challenge, using immunofluorescence for reading parasitised RBC percentage on Acridine Orange-stained slides [17].

### CD Analysis

Peptide structures in solution were acquired by circular dichroism measurement in water and 30% TFE mix. The spectra were obtained on a JASCO J-810 spectrometer at room temperature. Data was assessed at 190 to 260 nm wavelength using 20 nm/min scan rate and 1 nm band with. The data was collected using Spectra Manager Software and analysed using SELCOM3, CONTILL and CDSSTR database [19].

### NMR Spectroscopy

8 or 10 milligrams of each peptide (6583, 6584 and 6622) were dissolved in 500 µl TFE-d3/H20 (30/70 v/v). The basic NMR structure determination protocol [20] was as follows: proton spectra were assigned by DQF-COSY, TOCSY and NOESY; TOCSY and DQF-COSY spectra were then used to identify individual spin systems (amino acids) and NOESY (400 ms mixing time) was used for determining peptide primary and secondary structure. TOCSY spectra recorded at different temperatures (285–315 K) were used to obtain amide temperature coefficients for predicting hydrogen bonds (-ΔδHN/ΔTppb/K), as thoroughly described beforehand [14], [21].

### Structural Calculation

Peptide structure was determined by Accelrys software. NOE peaks, selected from 400 ms NOESY data sets, were integrated and converted into distance restraints. These restraints were grouped as strong, medium and weak (1.8–2.5 Å, 2.5–3.5 Å, and 3.5–5.0 Å distance restraints, respectively). Hydrogen bond constraints were introduced for slow exchange rate peptide NH, distance ranges involving likely NH–O hydrogen bonds were set at 1.8–2.5 Å. A family of 50 structures was obtained using Distance Geometry (DGII) software and then refined using simulated annealing protocol (DISCOVER software) to select those having reasonable geometry and fewer violations.

### HAPB Superimposition on Crystallised DBL Protein Fragments

The 3D structure of DBL domains from PDB 2XU0 [22], 3CML [23] and 2WAU databases [9] was used for selecting peptide regions presenting high activity binding peptides (HABP) based on aminoacid sequence alignment between strains. InsightII biopolymer molecular software (Accelrys Inc.) was used for such superimposition using backbone superimposition based on RMSD criteria as well as H-bond measurement between HABPs forming the niche which is important for binding site receptors.

## Results and Discussion

**Figure 1:**
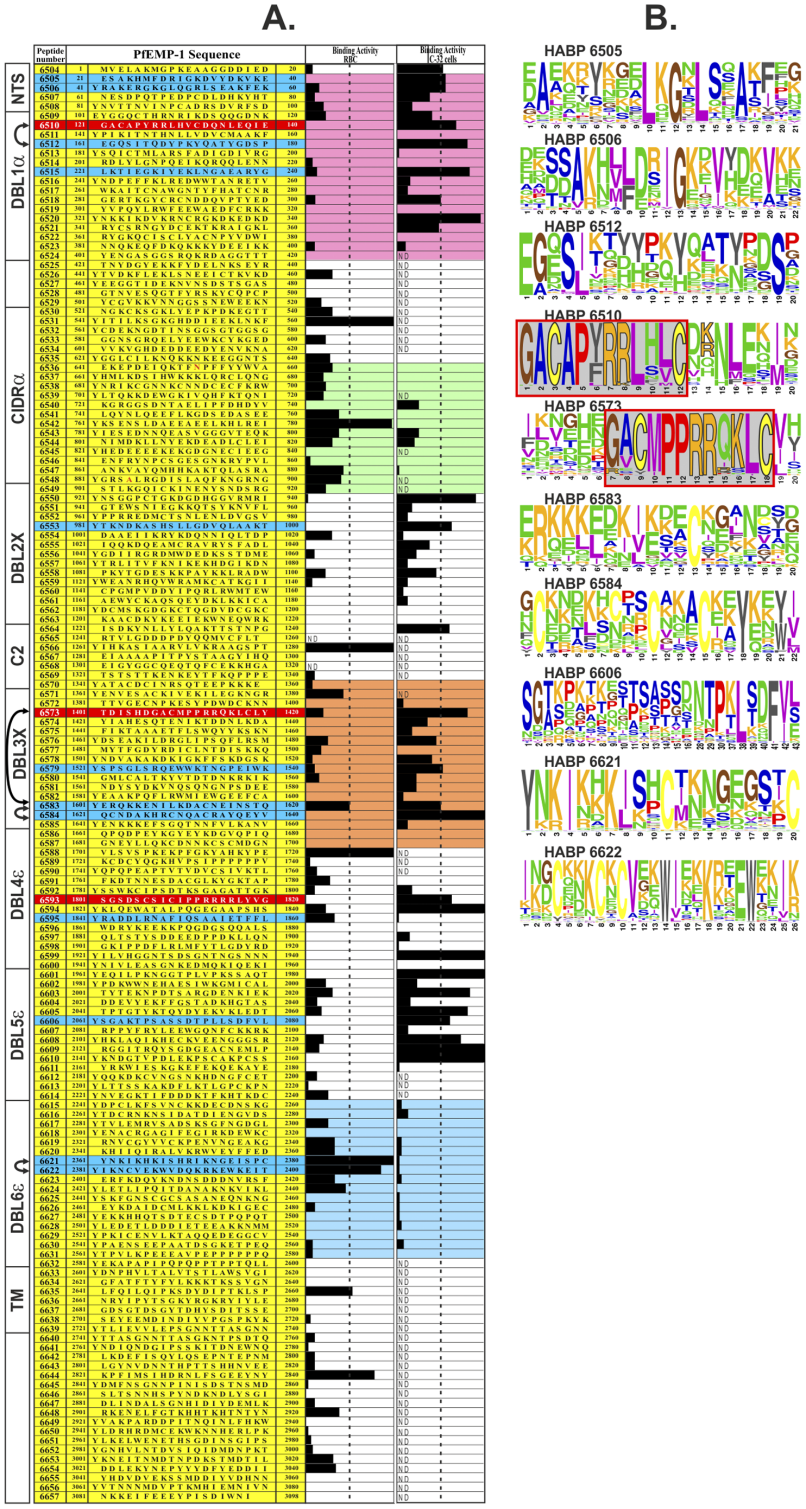
Identification of *Pf*EMP1 HABPs and variability sequence between *Plasmodium falciparum* strains. (**A**) Dd2 *Pf*EMP1-based amino-acid sequence synthetic peptides’ RBC and C32 cell binding activity (black bars represent specific binding activity slope); above 2% (dotted line) were considered HABPs [11]–[14]. Blue shows HABPs chosen for immunization and red those containing canonical or homologous (GACxPxRRxxLC) binding motif. Left, schematic representation of *Pf*EMP1 domains showing H-bonds between HABPs (arrows); head structure recombinant fragments containing NTS and DBL1α (fuchsia), CDR1α (green), DBL3X (orange) and DBL6ε (blue), 3D structure determined by X-ray crystallography. (**B**) Sequence logos for amino acid conservation in corresponding HABPs according to their frequency in >100 strains; each amino acid height reflects their relative frequency (%) and thus their contribution to conservation.

One hundred and fifty 20-mer long peptides were synthesised using the Dd2var1 clone *Pf*EMP1 amino acid sequence, finding 25 HABPs able to bind specifically to C32 endothelial cells (amelanotic melanoma-derived) and 10 O+ red blood cell (RBC) binding HABPs (Figure 1A). Twelve C32 HABPs and two RBC HABPs were randomly selected for being modified as mHABPs [12]–[14].

Ninety-two mHABPs (synthesised using Dd2 sequence, Indochina) were used for immunising groups of four to ten *Aotus* monkey groups per mHABP, since *Aotus* immune system is similar to that of humans (90%–100% identity) [24]. Immunogenicity was determined by immunofluorescence antibody test (IFA) using the FCB-2 strain (Colombia) and reactivity by Western blot (WB) using FVO strain (Vietnam) IE lysate. mHABP protection-inducing ability was determined following 2^nd^ or 3^rd^ immunisation by intravenously inoculating 100,000 fresh IE from other *Aotus* previously infected with the heterologous *Aotus-*adapted FVO strain [12]–[14].

**Figure 2:**
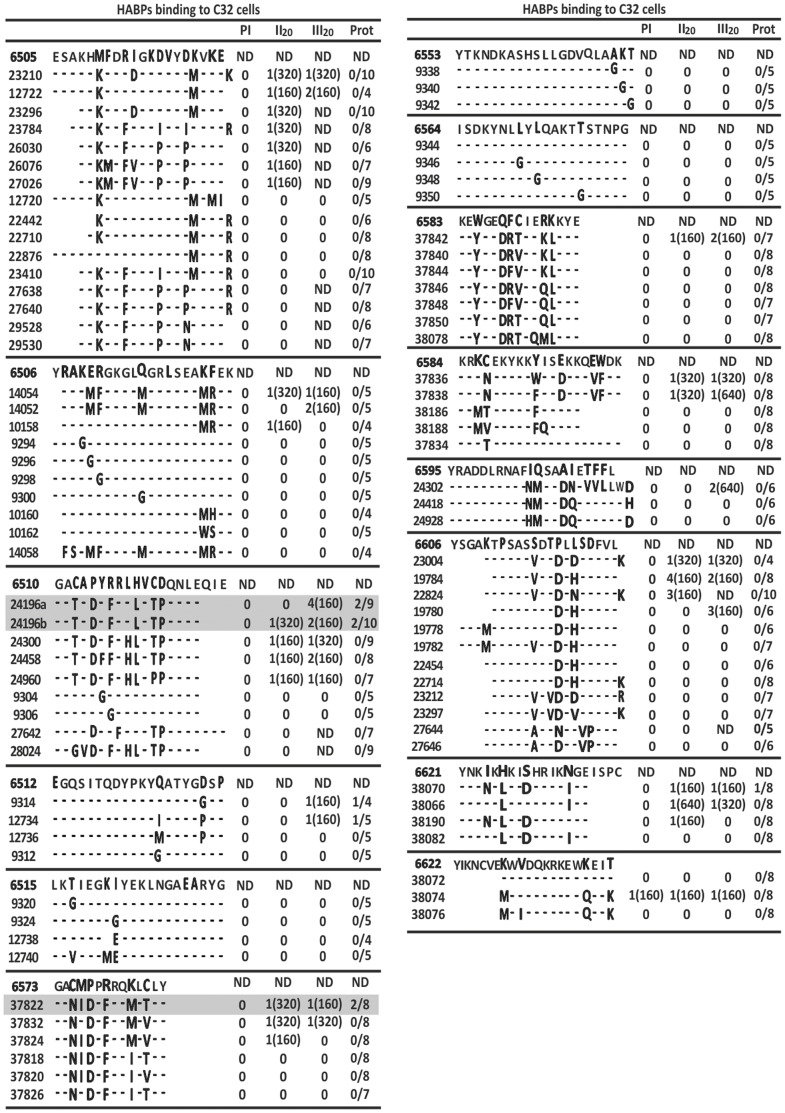
Humoral immune response and protective efficacy induced by *Pf*EMP1 HABPs derived peptides in *Aotus* monkeys. *Aotus* monkeys’ humoral immune responses and protective immunity induced by *Pf*EMP1-derived peptides, according to our serial numbering system with corresponding amino acid sequence (modifications in bold). Reciprocal IFA antibody titres in bleeding 20 days after second (II_20_) and third (III_20_) immunisation and number of protected monkeys in experimental challenge [12], [14].

**Figure 3:**
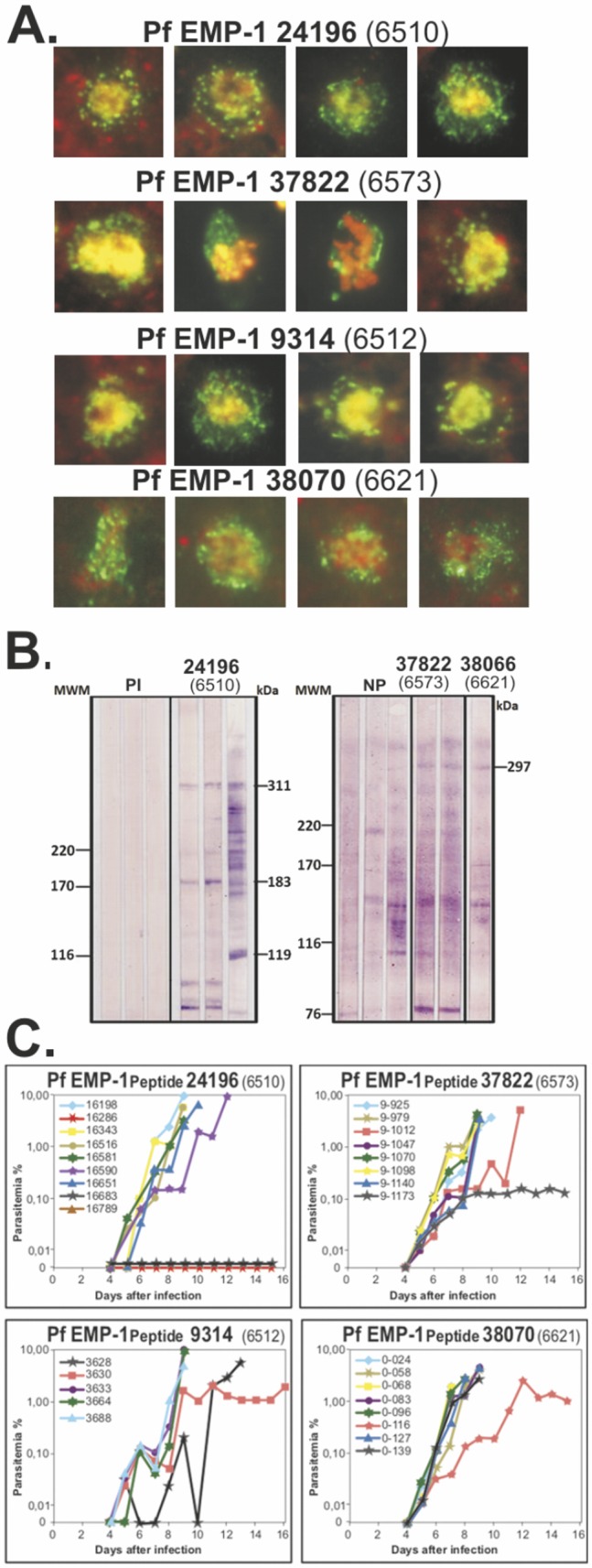
Immunological assessment in animal model trials using modified HABPs. (**A**) IFA assay showing characteristic *Pf*EMP1 dotted pattern on IE membrane, using sera from immunised *Aotus* monkeys, corresponding mHABP number on top. (**B**) WB recognition of ∼300 kDa protein in IE lysate from mHABP-immunised *Aotus* sera. PI: preimmune sera; NP: non-protected. (**C**) Comparative course of parasitaemia in *Aotus* immunised with mHABPs. Note the complete absence of parasites (full protective immunity) induced by 24196 (6510) in the first trial; the second with 10 monkeys gave similar results.

Around ∼30% mHABPs induced high IFA titres (1∶160–1∶640) (Figure 2 and 3A), thus demonstrating transcontinental strain-transcending antibody (Ab) induction, even though most did not induce any protection against experimental challenge (Figure 2).

Strikingly, mHABP **24196 (**GA**T**A**D**Y**F**RL**L**V**TP**QNLE) derived from 6510 (GACAPYRRLHVCDQNLEQIE) (mHABP numbers and their modifications shown hereafter in bold), containing the GACxPxRRxxLC motif (Figure 1B), induced high Ab titres (≥1∶160) in 4/9 *Aotus* as assessed by IFA (Figure 2); WB recognised a 310 kDa protein similar to *Pf*EMP1 molecular weight (Figure 3B). Two *Aotus* became fully protected against experimental challenge (0 parasites in blood) throughout the whole experiment (Figure 3C) and 2/10 from a new group of *Aotus* monkeys became totally protected (the same ones displaying high IFA titres and reacting with the ∼310 kDa band (by WB), demonstrating that **24196** induced strain-transcending fully protective immunity in 4/19 (∼21%) monkeys. **24196** (GATADY**F**RL**L**V**T**PQ**N**LE) displayed a characteristic HLADRβ1*0405 binding motif and binding register (highlighted in grey), an allele found with similar frequency in humans and *Aotus* (∼21%) [24].

6510 did not induce antibodies in animal immunizations [25], [26] and antibodies raised against recombinant proteins containing this sequence have not recognised 6510, nor African human immune sera [27], confirming 6510’s immunological silence [12]–[14].

Two of the eight *Aotus* immunised with **37822** (GAN**I**DP**F**R**Q**ML**T**LY) derived from 6573 (GACMPPRRQKLCLY), also containing the GACxPxRRxxLC motif (Figure 1B) (underlined) localised in DBL3X, developed high Ab titres (≥1∶160) (Figure 2) and was partially protected, parasitaemia being maintained at around ∼0.1% throughout the experiment (Figure 3C). **37822** displayed HLADRβ1*1501 binding motifs and registers (grey) having similar frequency in *Aotus* (∼15%) and humans.

Such striking data showed that the canonical GACxPxRRxxLC motif localised in HB4 (≡HBb) [4]–[6], in the “head structure”, is the critical sequence inducing strain-transcending full protective immunity when appropriately modified, as in **24196** (6510), whilst **37822** (modified from homologous 6573) localized in DBL3X induced partial protective immunity. A homologous sequence (PxRRxxxC) present in DBL4ε domain is contained in 6593 (not used for immunisations) (Figure 1A) and a shorter PxRRxxLx sequence was found in DBL6ε N-terminus in some strains [28], confirming this motif’s presence in nearly all DBL domains [5].

Interestingly, highly-immunogenic mHABPS, like **9314** (EGQSITQDYPK**Y**QA**T**Y**G**GS**P**) derived from 6512 (EGQSITQDYPKY**Q**ATYG**D**S**P**) and **38070** (YNKNK**L**KI**D**H**R**IK**I**GE) derived from 6621 (YNK**I**K**H**KI**S**HRIK**N**GEISPC), also induced high antibody titres and partial protective immunity (parasitaemia being maintained at ∼1.5% in 1/8 monkeys each mHABP throughout the experiment, Figure 3C), similar to semi-immune African-adults, suggesting partial strain-transcending immunity, surpassed by tremendous polymorphism. **9314** and **38070** had perfectly classical HLADRβ1*0101 and HLADRβ1*0301 binding motifs and registers, respectively (grey).

IE usually express only one *Pf*EMP1 at a time but the parasite switches *var* gene expression, by a mechanism involving a *var* intron re-localization regulated by an 18 bp nuclear binding element that regulates actin polymerization [29] and leads to the change in host-cell receptor specificity and serotype [30], [31], evading the immune response [32]. Such polymorphism could partly explain the partial protective immunity obtained, despite mHABPs being properly modified [12] and high antibody titres being induced (Figure 2) but it has been also demonstrated that *Pf*EMP1 specifically induces a large panel of immune suppression mechanisms among these the early production of human γ interferon [33], but the domain (s) involved in such scape mechanisms remains to be identified.

### Strain-transcending Protection-inducing mHABP 3D Structure

**Figure 4:**
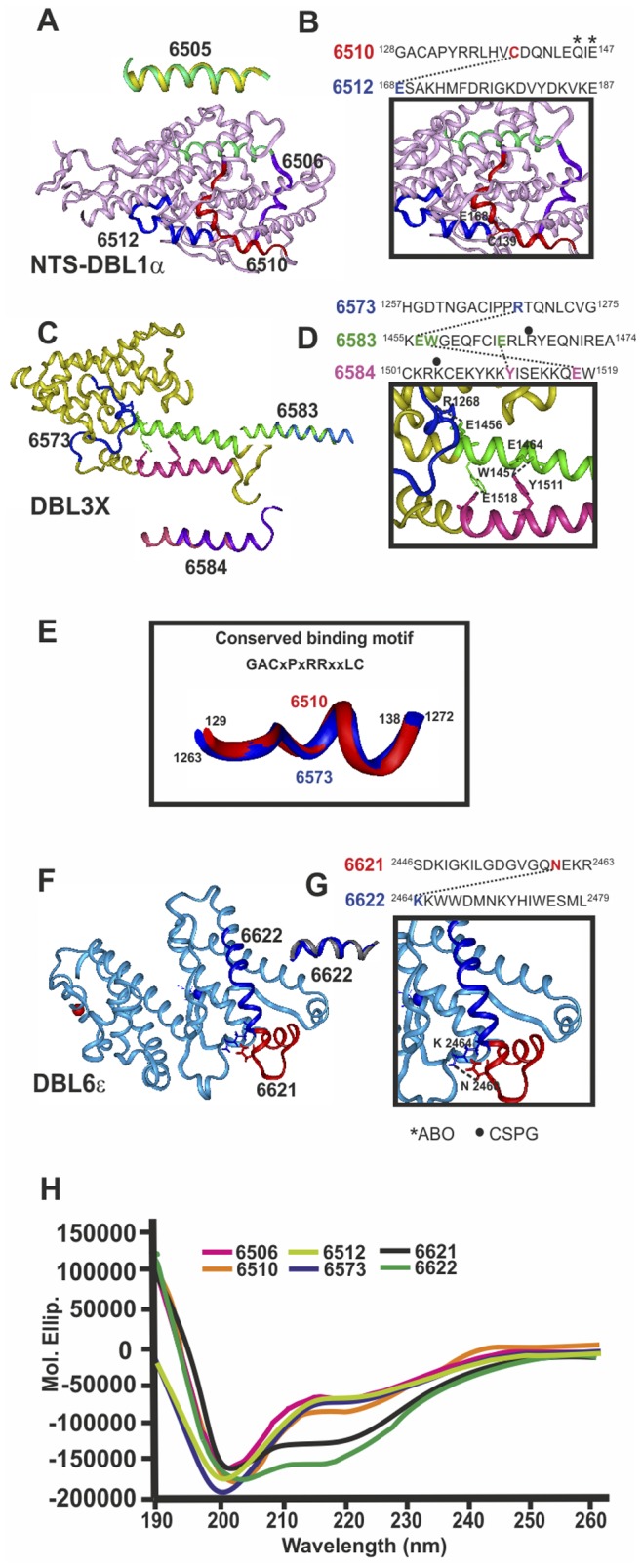
Structural characterization of HABPs present in crystallized Duffy binding like domains (DBL). DBL domain 3D structure determined by X-ray crystallography A) Head structure: DBL1α (PDB 2XU0) (pink), C) DBL3X (PDB 3CML) (yellow), F) DBL6ε (PDB 2WAU) (pale blue). ^1^H-NMR-determined structure localisation, displaying the perfect fit of HABP 6505 (yellow) superimposed onto DBL1α, 6583 (dark blue) and 6584 (purple) onto DBL3X and 6622 (grey) onto DBL6ε. B, D, G). H-bonds between HABP residues and their corresponding sequence on top, displaying relevant residues in binding to A blood group trisaccharides and CSPG (asterisk and black dot, respectively). E) Superimposed conserved binding motif fragments from 6510 and 6573. H) CD spectra for corresponding HABPs.

The 3D structure of the rosette-forming blood group A-binding Palo Alto VarO strain (Uganda) “head structure” containing the NTS-DBL1α-CIDR1γ region [34], the A4 strain (Brazil) chondroitin-sulphate-proteoglycan (CSPG) binding DBL3X domain [23], [35] and the 3D7 strain (unknown origin) DBL6ε domain binding to CSA has been determined by X-ray crystallography [9].

(reminder: our HABPs are based on Indochina Dd2 sequence). Our group determined that native HABP 6505 displayed a perfect α-helix structure by ^1^H-NMR (Figure 4A, yellow) [14]; when superimposed onto the NTS-DBL1α 3D structure (Figure 4A, green) it gave a 0.16 rmsd and 6583 and 6584 (Figure 4B, fuchsia and dark blue) had α-helix conformation (Figure S1 and Table S1), giving 0.43 and 0.52 rmsd, respectively, when superimposed onto the DBL3X sequence [23], [35]. It has thus been thoroughly demonstrated that chemically-synthesised HABPs display the same 3D structure as biologically-derived recombinant proteins [11], [12].

Circular dichroism (CD) revealed that 6621 and 6622 had a helical structure (Figure 4H), while SELCON3 deconvolution analysis revealed 0.785, 0.836 and 0.898 turn and unordered structure composition for 6510, 6512 and 6573.

6510 (located in the NTS-DBL1α fragment subdomain S1 between cysteine 3 and 4) contained a short αH1 helix and short 3_10_ H1 helix; partially unordered HABP 6573 (DBL3X subdomain S1) only displayed a short α-helix [23], the rest being unordered.

Thus native 6510 and 6573, parents of strain-transcending protective immunity inducing **24196** and **37822**, respectively, containing the GACxPxRRxxLC motif, displayed an almost completely unordered and similar structure in DBL1α and DBL3X since 6510 superimposition onto 6573 gives 0.65 rmsd (Figure 4E) explaining in part the cross protective immunity; in sharp contrast with strain-transcending non-protective antibody-inducing HABPs having helix structures (6505, 6506, 6583,6584 and 6622) (Figure 4A, C, F) and partially protection inducing 6512 (unordered) and 6621 (α helical and partially unordered, by CD and X-ray crystallography), suggesting an association between structure and immunogenicity and protection [12]–[14].

### Modifying H-bond-establishing Residues among cHABPs Induced Strain-transcending Immunity

3D analysis of 6510 (^128^GACAPYRRLHV**C** ^139^DQNLEQ*IE*^147^) showed that C139 HN established an H-bond with Oε_1_ from E168 present in 6512 HABP N-terminus (^168^ **E**GQSITQDYPKYQATYGDSP^187^) forming the niche where the A1 blood group terminal α-1,3 linked N-acetylgalactosamine (GalNAc) [34] bound through residues Q145 and E147 (asterisk in Figure 4B) [34], suggesting that modifying these H-bond-establishing residues among cHABPs via T139C replacement in **24196** was fundamental [12] for inducing fully-protective, strain-transcending antibody immunity (Figure 2 and 3C). Antibodies against these mHABPs might thus have been blocking IE to UE for rosette formation, thereby impeding IE agglutination and microvascular obstruction, associated with CM, making **24196** essential for severe malaria control in some individuals, as will be discussed later on.

By the same token, 6573 (^1257^HGDTNGACIPP**R** ^1268^QTQNLCVG^1275^), containing also conserved binding motif GACxPxRRxxLC (Figure 1A), established an H-bond between R1268 HNε and E1456 Oε2 present in 6583 (^1455^K**E** ^1456^ **W** ^1457^GEQFCI**E** ^1464^RL**R^•^**YEQNIRE^1474^); 6583 established another H-bond between E1464 Oε1 with OH in 6584 Y1511 (^1501^CKR**K^•^**CEKYKK**Y** ^1511^ISEKKQ**E** ^1518^W^1519^) to form a tripartite binding site for CSPG (Figure 4D, dot on top). Replacing 6573 R1268 by F in **37822** (^1262^GA**NID**P**F** ^1268^RQ**M**L**T**LY^1275^) induced strain-transcending immunity, controlling parasitaemia at <1% throughout the experiment, due to these cHABPs’ tremendous genetic variability means that blocking this highly polymorphic CSPG binding site could be relevant for PAM control and other severe malaria-associated problems where CSPG is involved.

Two HABPs were localised in DBL6ε, consisting of 7 variable blocks (VB) having limited polymorphism. Completely 3_10_-helix structure 6622 (^2464^ **K**KWWDMNKYHIWESML^2479^) determined by ^1^H-NMR (Figure 4F, grey ribbon) is localised in VB4; partially α-helical 6621 (^2446^SDKIGKILGDGVGQ**N** ^2460^EKR^2463^) (Figure 4F, red ribbon) localised in one of the elbows of DBL6ε domains, in VB4 [28], [36], established a H-bond between N2460 O (6621) and K2464 HN (6622), forming a niche for a non-identified RBC receptor binding (Figure 4G).

D2456S replacement in **38070** (6621) induced high Ab titres and partial protective immunity (∼1.5% parasitaemia), again confirming inter-HABP H-bond breaking’s relevance in immune induction. **38070** displayed binding motifs and registers (grey) characteristic of HLADRβ1*0301 allele (YNKNK**L**KI**D**H**R**IK**I**GE), an allele found ∼15% frequency in monkeys and humans.

6622-derived **38074** (MW**V**DQ**K**R**K**EW**Q**EIK), inducing non-protective antibodies, displayed the HLADRβ1*0802 binding motif and register (grey); this HABP is in highly polymorphic region (Figure 1B).

Recent studies have found predominant transcription of domain cassette DC8 (UPSB promoter followed by NTSβ-DBLα2-CIDRα1-DBLβ12-DBLγ4) and DC13 (encoding DBLα1.7-CIRDα1.4) (both containing the GACxPxRRxxLC motif) in blood samples from 70% of 88 Tanzanian children suffering severe malaria. This stresses the importance of **24196** (containing this motif) in inducing strain-transcending complete protective immunity against severe malaria in HLADRβ1*04 individuals, suggesting that more HABPs from other IE membrane-expressed molecules (like histidine-rich protein-II-derived **24230** (6800) under HLADRβ1*07 control [11]–[14], [37], STEVOR [38], [39]; RIFIN etc.) are needed to obtain definitive full protection against severe malaria.

These large functional-structural and immunological studies show that strain-transcending complete protective immunity against severe malaria can be fulfilled through previously defined principles [11]–[14] modifying the GACxPxRRxxLC conserved motif (canonical in the *Pf*EMP1 “head structure”) binding to endothelial cells. This, in turn, leads towards a fully-protective, multi-epitope, multi-stage, minimal subunit-based, chemically-synthesised definitive antimalarial vaccine [11]–[14].

## Supporting Information

Figure S1 Summary of sequential and medium range NOEs of 6583, 6584 and 6622. Summary of sequential and medium range NOEs determined in H2O/TFE-d3 (70%/30%). NOE intensity is indicated by bar height. The numbers inside the diagram are the 3J coupling constants. Δ represents residues involved in an H-bond.
(TIF)

Table S1 Summary of 6583, 6584 and 6622 structure calculation.
(DOCX)
